# Implementation of feasible health care services for smoking cessation in persons with mental illness – barriers and facilitators

**DOI:** 10.1192/j.eurpsy.2025.151

**Published:** 2025-08-26

**Authors:** A. Høye

**Affiliations:** 1 Department of Clinical Medicine, UiT The Arctic University of Norway; 2 Division of Mental Health and Substance Abuse, University Hospital of North Norway, Tromsø, Norway

## Abstract

**Introduction:**

Smoking remains a significant cause of mortality and morbidity, with a higher prevalence among those with severe mental illness compared to the general population. Patients with psychotic disorders are the most frequent smokers, which contributes to increased somatic diseases and cardiovascular risk. Existing guidelines for smoking cessation are not well-suited for patients with mental disorders, and these patients often lack confidence in their ability to quit. An effective intervention must therefore be comprehensive and interdisciplinary to increase motivation. As part of the national, Norwegian multicenter project **“**Preventing Multimorbidity in Severe Mental Disorders with a Multidisciplinary Approach (CVD-MENT), the subproject *User-Driven Smoking Cessation Group* has been launched. The idea of users’ active participation as co-leaders of a smoking cessation group comes from patients and is based on their experiences with their own smoking cessation attempts.

**Objective:**

To investigate whether a new, tailored treatment specifically targeted at patients with severe mental disorders affects smoking behaviour in a patient group known to have a very high risk of cardiovascular disease.

**Materials and methods:**

The program for the User-Driven Smoking Cessation Group is based on the existing template for smoking cessation groups described by the Norwegian Directorate of Health, but is adapted to the patient group. The study is conducted at three Norwegian hospitals. International recommendations for smoking cessation counselling describe the approach to unmotivated and ambivalent participants in five R’s, all of which are emphasized in the project:

1. Relevance - Help the patient reflect on whether tobacco cessation might be relevant
2. Risks - Discuss personally related health risks
3. Rewards - Help the patient articulate potential benefits of quitting tobacco
4. Roadblocks - Identify barriers preventing the patient from attempting to quit
5. Repetition - Prepare the patient for discussing this again at the next meeting

The project has developed a program for open smoking cessation groups specifically tailored for individuals with severe mental disorders. Patients who have successfully quit smoking actively participate in leading the group alongside healthcare professionals. The content focuses on smoking cessation, but other factors known to impact cardiovascular risk are addressed in the semi-structured teaching over seven sessions. Training is provided for users who are co-leaders.

**Conclusion:**

Experiences from a pilot study and preliminary experiences and results from the first groups will be presented and discussed.

**Disclosure of Interest:**

None Declared

